# Effects of workload and saddle height on muscle activation of the lower limb during cycling

**DOI:** 10.1186/s12938-024-01199-y

**Published:** 2024-01-16

**Authors:** Fangbo Bing, Guoxin Zhang, Yan Wang, Ming Zhang

**Affiliations:** 1 Department of Biomedical Engineering, Faculty of Engineering, The Hong Kong Polytechnic University, Hong Kong SAR, 999077 China; 2 Research Institute for Sports Science and Technology, The Hong Kong Polytechnic University, Hong Kong SAR, 999077 China; 3 The Hong Kong Polytechnic University Shenzhen Research Institute, Shenzhen, 518057 China

**Keywords:** Electromyography, Cycling workload, Saddle height, Lower limb, Muscle activation, Muscle coordination

## Abstract

**Background:**

Cycling workload is an essential factor in practical cycling training. Saddle height is the most studied topic in bike fitting, but the results are controversial. This study aims to investigate the effects of workload and saddle height on the activation level and coordination of the lower limb muscles during cycling.

**Methods:**

Eighteen healthy male participants with recreational cycling experience performed 15 × 2-min constant cadence cycling at five saddle heights of 95%, 97%, 100%, 103%, and 105% of greater trochanter height (GTH) and three cycling workloads of 25%, 50%, and 75% of functional threshold power (FTP). The EMG signals of the rectus femoris (RF), tibialis anterior (TA), biceps femoris (BF), and medial gastrocnemius (MG) of the right lower limb were collected throughout the experiment.

**Results:**

Greater muscle activation was observed for the RF and BF at a higher cycling workload, whereas no differences were observed for the TA and MG. The MG showed intensified muscle activation as the saddle height increased. The mean and maximum amplitudes of the EMG signals of the MG increased by 56.24% and 57.24% at the 25% FTP workload, 102.71% and 126.95% at the 50% FTP workload, and 84.27% and 53.81% at the 75% FTP workload, respectively, when the saddle height increased from 95 to 100% of the GTH. The muscle activation level of the RF was minimal at 100% GTH saddle height. The onset and offset timing revealed few significant differences across cycling conditions.

**Conclusions:**

Muscle activation of the RF and BF was affected by cycling workload, while that of the MG was affected by saddle height. The 100% GTH is probably the appropriate saddle height for most cyclists. There was little statistical difference in muscle activation duration, which might be related to the small workload.

**Supplementary Information:**

The online version contains supplementary material available at 10.1186/s12938-024-01199-y.

## Introduction

Cycling is a popular activity for sports, recreation, and transportation. It involves repetitive movements of the lower limbs, which can lead to muscle fatigue and non-traumatic injuries of joints if proper techniques and equipment are not used [1]. Saddle height is an essential factor affecting cycling performance and muscle activation patterns [1]. Currently, there are many criteria for defining the appropriate saddle height, such as body height, leg length, joint angle, and joint range of motion [2]. The most popular approach is still proprioception and anthropometric methods because of the complicated process of dynamic bike fitting [3]. Additionally, the cycling workload described in watts of power also influences muscle activation patterns, and it can be manipulated through gear ratio selection or a cycling platform [4].

The activation pattern of the lower limb muscles is related to the mechanical work required during the cycling movement [5]. For instance, the relaxation and contraction patterns of the quadriceps femoris, biceps femoris (BF), hamstrings, and gastrocnemius muscles directly affect the knee joint’s ability to flex and extend. An outdoor cycling experiment found that workload and mechanical efficiency significantly affected the coordination pattern of the rectus femoris (RF) and vastus lateralis (VL) muscles [6]. However, cycling workload and cadence are uncontrollable in outdoor experiments, which could also result in faulty quantitative analyses. Experiments conducted in laboratories showed that pedal power level, that is, cycling workload, had little impact on the pattern of surface electromyography (EMG) signals of the main lower limb muscles [7]. The power in Hug et al.'s experiment [8] was less than 125 W, and the EMG signal of the gastrocnemius did not change as the power changed. In another experiment, they found that the EMG threshold of VL appeared at 75–80% of peak power output [9]. As the power level was further increased, the EMG signal displayed a non-linear sharp increase. However, no similar rule was found in the RF, semimembranosus, or BF. When the cycling workload increased from 60 to 100% of the maximum aerobic power, the EMG signals of the vastus medialis (VM), VL, and RF were dramatically changed [10]. During 30 s of sprint cycling, substantial power reduction was accompanied by a decrease in EMG amplitude for the gluteus maximus, gastrocnemius, VL, and RF, and all of these muscles showed later onset and earlier offset [11]. The association between knee extensor isometric force and power production was much clearer during the extension period than during the full crank cycle. However, a subperiod EMG study has not been conducted, which is important to explain the relationship between workload and EMG signals. In addition to activation amplitude and duration, exploring the effect on the onset/offset timing may be more beneficial in explaining the coordination patterns of the lower limb muscles.

The cycling position affects the synergy between different muscle groups of the lower limbs [12], so proper bike fitting is crucial for enhancing pedaling efficiency and evaluating proper cycling position. Saddle height was the most explored configuration parameter. The first reason is that saddle height is the most straightforward and popular setting to adjust to fit an individual’s leg length. The second reason is that changes in saddle height can significantly affect the extent of the muscle contractions of lower limbs. In this sense, the close relationship between saddle height and non-traumatic injuries such as knee pain and lower back pain has been confirmed by many studies [3, 13]. The duration of gastrocnemius and VL eccentric contractions decreased with increasing saddle height, which was set according to the trochanteric height, while the duration of BF significantly increased during a crank cycle [14]. However, only three heights were set, and the case in which the saddle height was higher than the trochanteric height was also not investigated. Studies have shown that the most comfortable saddle height is between 97 and 103% of the trochanteric height [15]. A reduced activation of gastrocnemius at lower saddle height was displayed, regardless of whether the saddle height was in the downwards or upwards position relative to the neutral position (106%–107% of crotch height) [16]. The cyclists also claimed that they felt more uncomfortable in the changed saddle heights than in the neutral position. However, this previous study only conducted a 10-min test and concentrated primarily on the comfort of the cyclists. In addition to choosing the height based on the length of the lower limbs, the joint angle is an alternative. According to the knee extension angles taken statically at the 6 o’clock crank position, the EMG of semitendinosus was greater as saddle height climbed from the 20° to the 40° position. However, only the normalized root-mean-squared EMG was provided in the previous study. More proof that saddle height impacts activation patterns of lower limb muscles is necessary to reveal the relationship between saddle height and cycling performance and injury risk.

The purpose of our study was to examine the activation of lower limb muscles, specifically RF, tibialis anterior (TA), BF, and medial gastrocnemius (MG), in response to various cycling settings with five saddle heights and three cycling workloads selected for individuals. It was hypothesized that the activation levels of different muscles did not change consistently as the saddle height increased, and the maximum value and mean value of normalized EMG increased as cycling workload increased.

## Results

The statistical analysis indicated that there were no outliers, and the residuals were normally distributed (*p* > 0.05). The homogeneity of variance test was satisfied, as assessed by Levene's test (*p* > 0.05). The results of Mauchly's test showed that the assumption of sphericity was violated (*p* < 0.05). Therefore, the separate one-way ANOVA with Greenhouse‒Geisser correction was carried out on the EMG mean and maximum using saddle height and workload chosen as the independent factors, respectively. The results show that the workload had a great influence on the mean of the EMG envelope of RF (F(2,207) = 3.053, *p* = 0.049, ${\eta }^{2}$ = 0.29) and BF (F(2,207) = 1.725, *p* = 0.181, ${\eta }^{2}$ = 0.016), while the saddle height only had an influence on the EMG mean of MG (F(4, 205) = 5.685, *p* < 0.0005, ${\eta }^{2}$ = 0.1). Similarly, the corresponding EMG maximum of the RF (F(2,207) = 8.108, *p* = 0.001, ${\eta }^{2}$ = 0.073) and BF (F(2,207) = 3.281, *p* = 0.04, ${\eta }^{2}$ = 0.031) were also affected by workload. The saddle height influenced the maximum EMG of the MG (F(4,205) = 9.819, *p* < 0.0005, ${\eta }^{2}$ = 0.161).

### Normalized EMG envelope

**Fig. 1:**
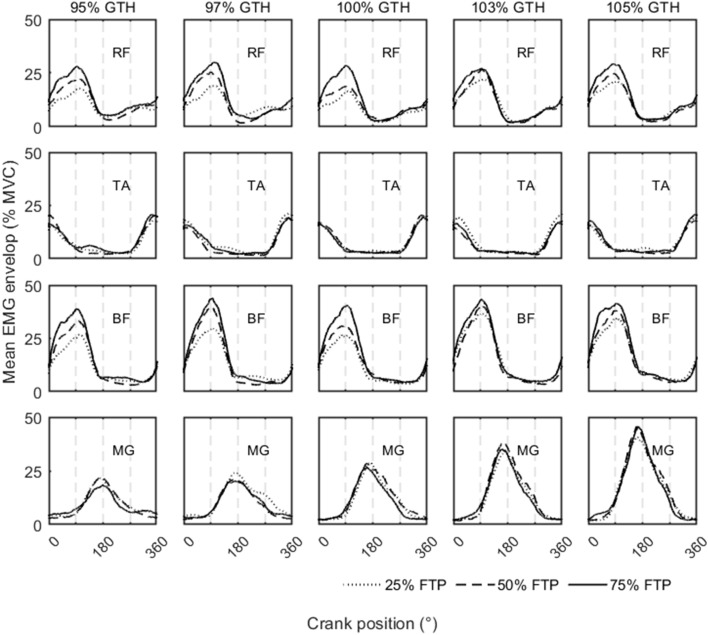
The average of normalized surface electromyography of lower limb muscles. EMG: electromyographic; RF: rectus femoris; TA: tibialis anterior; BF: biceps femoris; MG: medial gastrocnemius

The EMG envelope is shown in Fig. 1. The horizontal axis represents one cycle of cycling, from 0° to 360°. The vertical axis represents the level of EMG activation, normalized by MVC. The subfigures in the first row to the fourth row display the respective EMG envelopes of the RF, TA, BF, and MG muscles. Five saddle heights are represented in the first through fifth columns, which are 95%, 97%, 100%, 103%, and 105% of the GTH, respectively. In each subfigure, the effect of workload was compared for that muscle at a specific saddle height. The dotted, dashed, and solid lines represent workloads of 25%, 50%, and 75% of the FTP, respectively. For the RF and BF muscles, the EMG envelopes and the maximum increased significantly as the workload increased. The EMG activation was greatest when the workload was set at 75% FTP. However, the EMG activations of the TA and MG were almost unaffected by the workload. Comparing the five subplots for each muscle, the EMG envelope only increased with saddle height for the MG (fourth row from left to right), whereas saddle height had little effect on RF, BF, and TA.

### Mean and maximum of EMG signal envelope

**Fig. 2:**
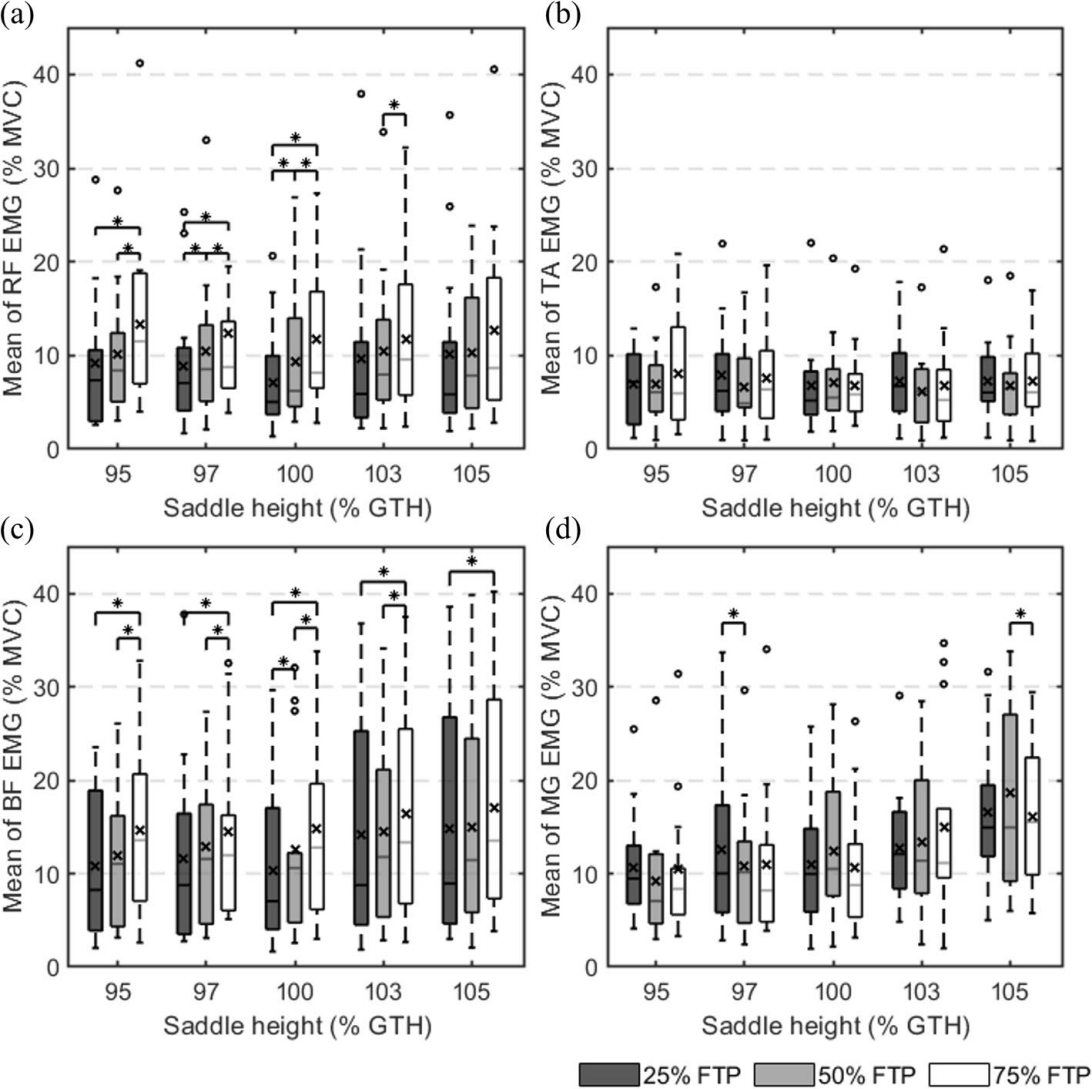
Mean values of the normalized surface electromyographic (EMG) envelope. **a** The mean EMG of rectus femoris; **b** the mean EMG of tibialis anterior; **c** the mean EMG of biceps femoris; **d** the mean EMG of medial gastrocnemius. ○ represents an outlier. * indicates a statistical difference (*p* < 0.05). × denotes the mean of the box values. FTP: functional threshold power; GTH: greater trochanter height; MVC: maximum voluntary contraction; RF: rectus femoris; TA: tibialis anterior; BF: biceps femoris; MG: medial gastrocnemius

**Fig. 3:**
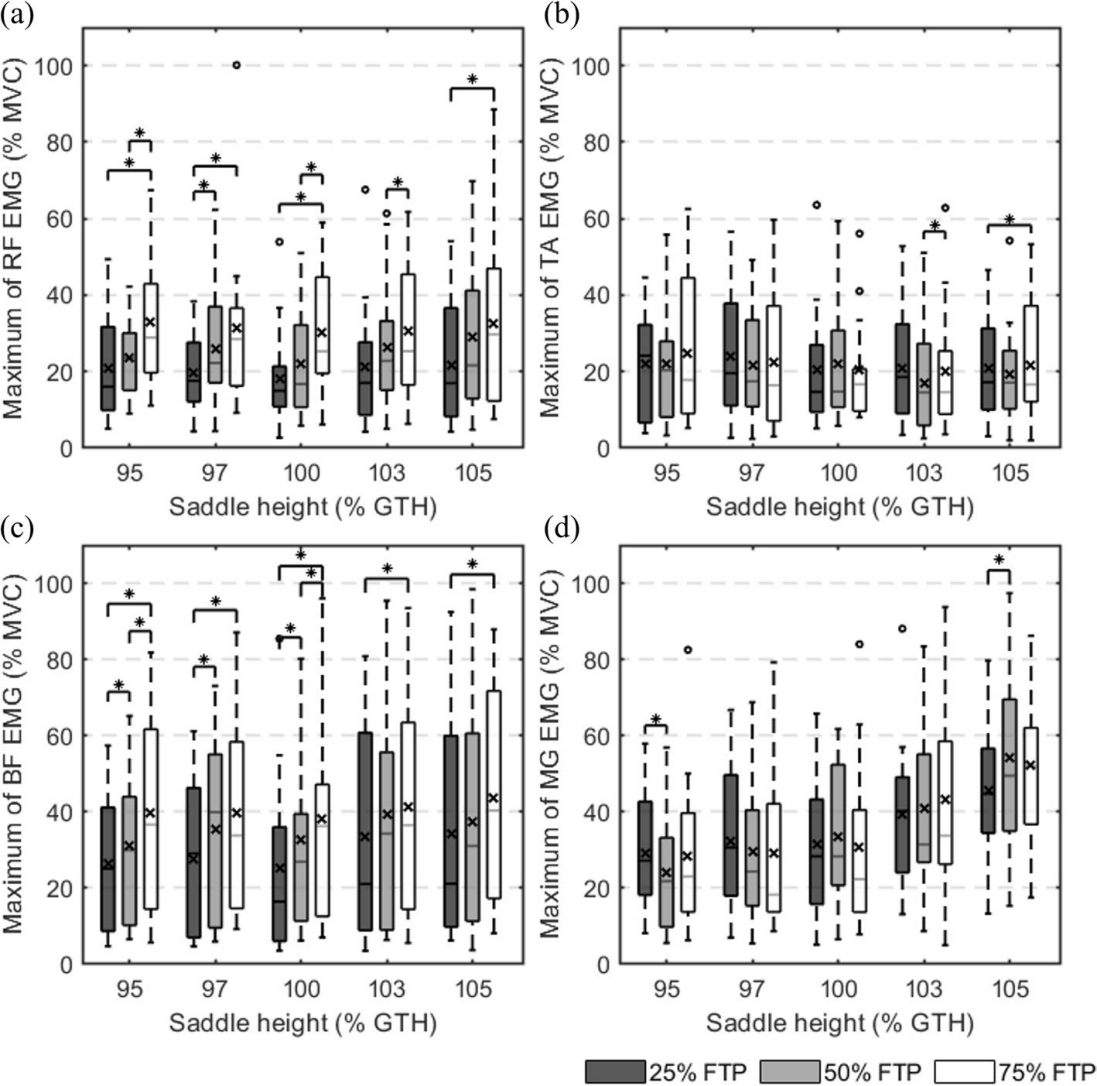
Maximum values of the normalized surface electromyographic (EMG) envelope. **a** The maximum EMG of rectus femoris; **b** the maximum EMG of tibialis anterior; **c** the maximum EMG of biceps femoris; **d** the maximum EMG of medial gastrocnemius. ○ represents an outlier. * indicates a statistical difference (*p* < 0.05). × denotes the mean of the box values. FTP: functional threshold power; GTH: greater trochanter height; MVC: maximum voluntary contraction; RF: rectus femoris; TA: tibialis anterior; BF: biceps femoris; MG: medial gastrocnemius

**Fig. 4:**
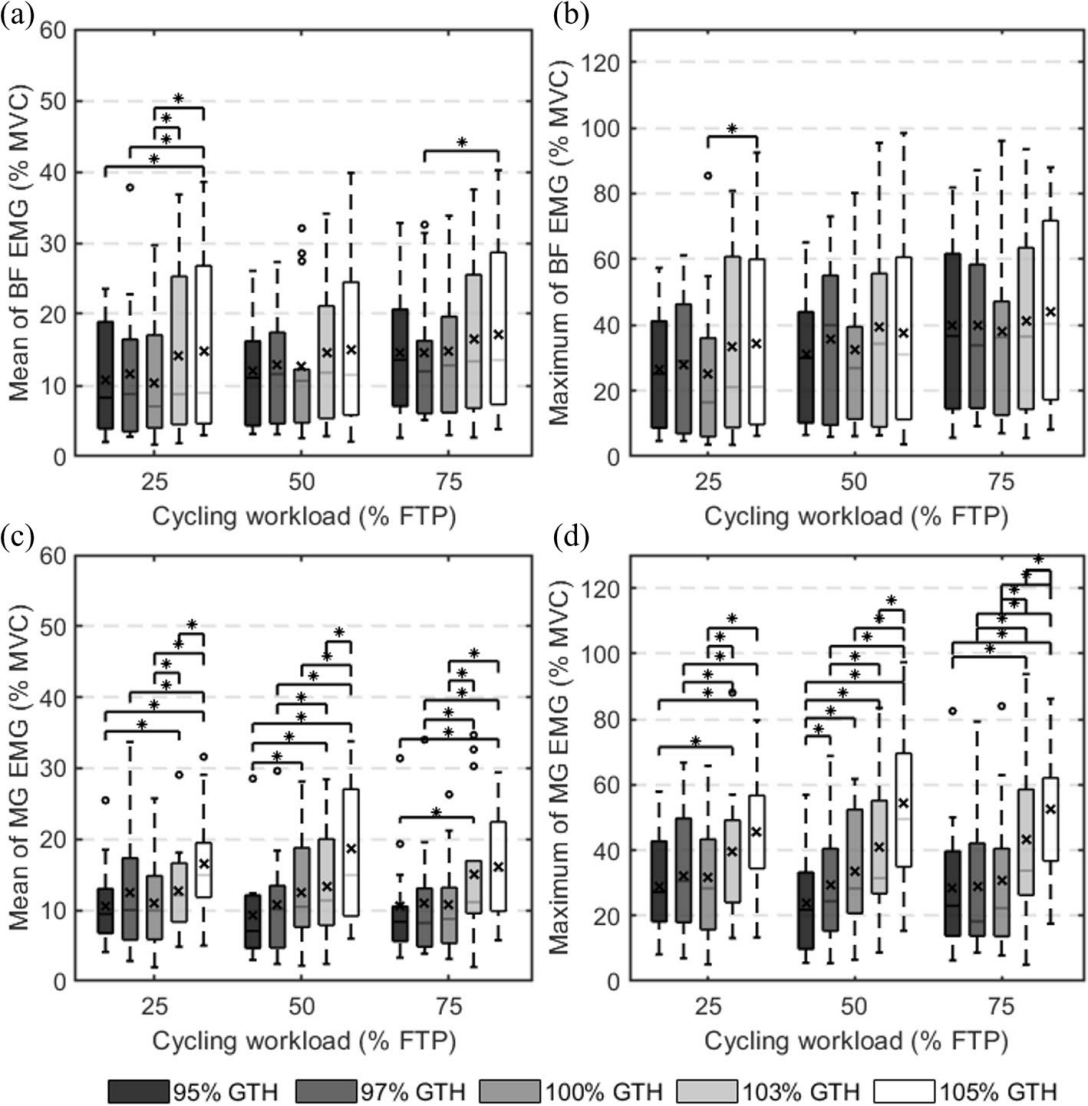
Mean and maximum values of the normalized surface electromyographic (EMG) envelope of the biceps femoris and medial gastrocnemius. **a** The mean EMG values of the BF; **b** the maximum EMG values of the BF; **c** the mean EMG values of the MG; **d** the maximum EMG values of the MG. ○ represents an outlier. * indicates a statistical difference (P < 0.05). × denotes the mean of the box values. FTP: functional threshold power; GTH: greater trochanter height; MVC: maximum voluntary contraction; RF: rectus femoris; TA: tibialis anterior; BF: biceps femoris; MG: medial gastrocnemius

The comparison of the mean and maximum values of the EMG envelope is presented in Figs. 2 and 3, and groups with statistically significant differences are denoted by asterisks (*). The x-axis represents the saddle height of 95%, 97%, 100%, 103%, and 105% of the GTH, and the y-axis represents the mean of the EMG envelope normalized to the individual MVC value (%MVC). Three cycling workloads were compared under each saddle height condition. Crosses on the boxes indicate the data’s mean. The specific mean and maximum values of the EMG envelope are summarized in the additional files (see Additional file 2: Table A1 and Additional file 3: Table A2). The saddle height had a statistically significant effect on the EMG results of the BF and MG but not the RF and TA. Therefore, the mean and maximum values of the EMG envelopes of the BF and MG at different saddle heights are presented separately in Fig. 4. The x-axis represents the cycling workload of 25%, 50%, and 75% of the individual FTP. The y-axis represents the normalized EMG values as same as in Fig. 3.

The EMG mean and maximum of RF are shown in Figs. 2a and 3a. The outcomes demonstrated that as workload increased at the same saddle height, the mean and maximum values increased. The increase in the mean with saddle height at 95% and 100% GTH showed a noticeable significant difference. The maximum EMG increased with increasing workload and exhibited a statistically significant difference regardless of the saddle height. In comparison to other heights, the EMG mean and maximum at 100% GTH saddle height were slightly lower.

Figures 2b and 3b display the EMG mean and maximum of the TA. The changes in cycling workload and saddle height had no appreciable impact on EMG activation in the TA. Except for an increase in workload from 25 to 75% FTP at 105% GTH and from 50 to 75% FTP at 103% GTH, there is a statistical difference.

The EMG mean and maximum of the BF are shown in Figs. 2c and 3c. Similar to RF, the EMG mean and maximum of the BF rose with the workload, and there were statistical differences in the comparison of workload with various saddle height circumstances. On the other hand, the saddle height had an effect on the EMG signals of the BF at a 25% FTP cycling workload, as shown in Fig. 4a and b. This effect disappeared when the workload was increased to 50% FTP and 75% FTP. It is worth noting that when the workload was 25% FTP and 50% FTP, the mean and maximum EMG activations of the BF were both minimal at the saddle height of 100% of the GTH.

Figures 2d and 3d demonstrate the EMG mean and maximum of the MG. Its EMG activation levels were less affected by workload, which was similar to that of the TA. Additionally, there is no consistent rule for the variation. However, the MG was most obviously affected by saddle height compared with other muscles. Figure 4c and d shows that the mean and maximum values of the EMG increased as the saddle height increased. When only the saddle height was considered as the independent variable without considering the workload, the mean EMG increased from 27.12 ± 16.88% to 50.76 ± 21.77% MVC with saddle height from 95% GTH to 105% GTH (MD = − 23.64, 95% CI [− 35.63, − 11.65], *p* < 0.001). All the mean and maximum EMG values increased more sharply when the saddle height increased from 100 to 105% GTH than from 95 to 100% GTH, indicating a rapid increase in muscle activation.

### Duration of muscle activation

**Fig. 5:**
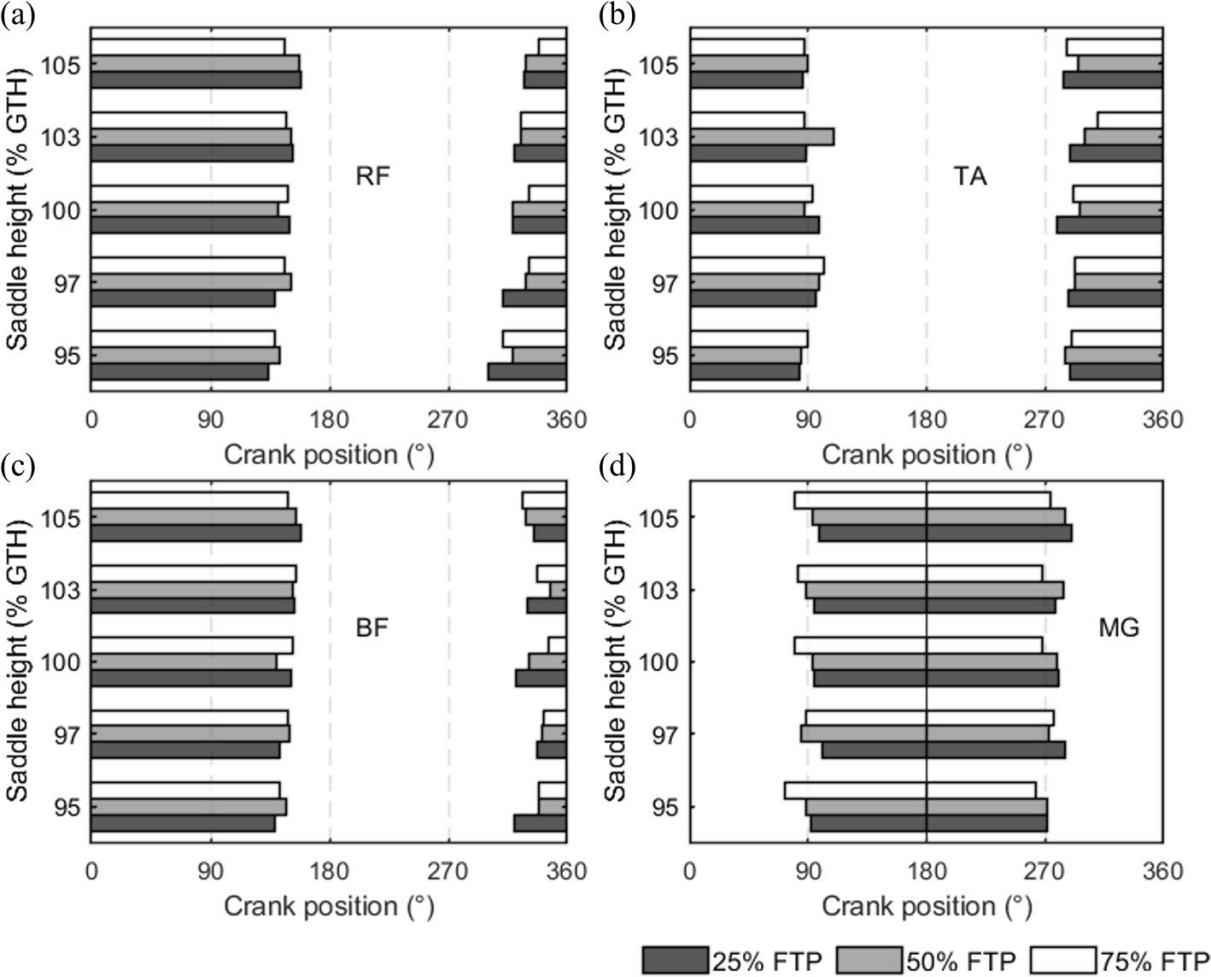
Relative mean onset, offset timing, and duration of muscle activation. **a** rectus femoris (RF), **b** tibialis anterior (TA), **c** biceps femoris (BF), and **d** medial gastrocnemius (MG)

Figure 5 shows the timing of onset/offset timing and duration of muscle activation for the RF, TA, BF, and MG based on the detection threshold. The x-axis represents the crank position (°), and the y-axis represents the saddle height of 95%, 97%, 100%, 103%, and 105% of the GTH. Table 1 displays the percentage of muscle activation time in the total time of the propulsive phase, recovery phase, and a complete crank cycle. The statistical differences in these times between different cycling workloads at each saddle height are summarized in Table 2. Most of the results were not statistically different. This suggested that both saddle height and workload have little effect on the onset and offset timing. For RF, the total duration of activation decreased as the workload improved, and the decrease in duration time started to be statistically different when the saddle height continued to grow from 100% GTH to 105% GTH. Except for the 97% GTH condition, the percentage of duration during the propulsive phase for the MG increased with the cycling workload. In particular, the significant difference was most evident at the saddle height of 105% GTH. In this case, the percentage of activation duration increased from 45.38 ± 11.19% to 48.10 ± 7.16% and then to 55.93 ± 12.89 (*p* = 0.009) as the workload increased from 25% FTP to 50% FTP and then to 75% FTP. The duration of activation during the recovery phase decreased with increasing workload for saddle heights of 95%, 100%, and 105% GTH. There was no consistent trend in the proportion of total duration across all cycling conditions.

**Table 1:** The percentage of muscle activation time in the total time

Saddle height (%GTH)	Workload (%FTP)	Rectus femoris			Tibialis anterior			Biceps femoris			Medial gastrocnemius		
		Propulsive (%)	Recovery (%)	A cycle (%)	Propulsive (%)	Recovery (%)	A cycle (%)	Propulsive (%)	Recovery (%)	A cycle (%)	Propulsive (%)	Recovery (%)	A cycle (%)
95	25	74.15 ± 15.32	33.38 ± 26.62	53.77 ± 10.64	46.18 ± 18.42	39.60 ± 14.32	42.89 ± 11.80	77.15 ± 18.02	22.44 ± 22.57	49.79 ± 8.19	49.13 ± 9.62	50.88 ± 8.19	50.01 ± 4.30
	50	78.96 ± 8.78	23.08 ± 23.26	51.02 ± 9.26	46.95 ± 17.48	41.76 ± 20.73	44.35 ± 12.84	82.05 ± 8.25	12.15 ± 14.18	47.10 ± 8.23	51.10 ± 16.83	50.78 ± 19.20	50.94 ± 6.86
	75	77.14 ± 16.75	26.82 ± 23.74	51.98 ± 8.03	49.55 ± 18.41	38.77 ± 12.51	44.16 ± 12.38	79.38 ± 19.25	12.14 ± 19.84	45.76 ± 1.10	59.46 ± 17.82	46.38 ± 12.21	52.92 ± 8.61
97	25	76.91 ± 16.72	26.73 ± 25.38	51.82 ± 10.70	52.98 ± 18.30	39.96 ± 19.40	46.47 ± 12.52	79.20 ± 12.92	12.62 ± 16.73	45.91 ± 7.18	43.87 ± 11.73	58.34 ± 17.68	51.10 ± 7.80
	50	83.74 ± 6.94	17.17 ± 19.86	50.46 ± 10.28	54.63 ± 25.79	37.56 ± 24.43	46.09 ± 11.77	82.97 ± 7.92	10.63 ± 18.30	46.80 ± 11.70	52.67 ± 13.11	51.28 ± 16.56	51.98 ± 7.70
	75	81.00 ± 6.10	16.06 ± 17.58	48.53 ± 9.27	56.53 ± 22.08	37.62 ± 23.18	47.07 ± 12.75	82.31 ± 9.91	9.63 ± 12.30	45.97 ± 5.90	50.54 ± 4.80	53.28 ± 13.41	51.91 ± 5.50
100	25	83.15 ± 10.41	23.21 ± 22.92	53.18 ± 8.98	54.61 ± 20.81	44.97 ± 23.33	49.79 ± 11.85	83.71 ± 8.72	21.87 ± 19.85	52.79 ± 9.57	47.38 ± 13.47	55.95 ± 10.65	51.66 ± 3.70
	50	78.12 ± 9.76	22.85 ± 22.19	50.49 ± 9.01	48.42 ± 12.57	35.08 ± 21.86	41.75 ± 8.80	77.66 ± 12.94	16.03 ± 18.64	46.84 ± 5.90	48.15 ± 16.03	55.00 ± 20.15	51.57 ± 5.47
	75	82.68 ± 7.81	16.04 ± 19.44	49.36 ± 9.14	51.68 ± 20.77	37.90 ± 20.09	44.79 ± 14.18	84.65 ± 7.58	7.90 ± 12.46	46.27 ± 6.33	55.39 ± 10.89	48.98 ± 8.87	52.19 ± 4.14
103	25	84.84 ± 11.77	22.44 ± 22.03	53.64 ± 9.04	49.46 ± 12.25	39.85 ± 15.18	44.65 ± 9.58	85.19 ± 10.64	16.78 ± 20.49	50.99 ± 8.58	47.61 ± 9.79	54.38 ± 13.58	51.00 ± 4.67
	50	83.83 ± 5.52	19.24 ± 18.13	51.54 ± 9.33	60.75 ± 22.02	33.52 ± 13.78	47.13 ± 13.66	84.66 ± 6.56	7.20 ± 9.08	45.93 ± 5.89	51.08 ± 10.64	57.56 ± 8.88	54.31 ± 4.51
	75	81.63 ± 13.62	19.43 ± 21.86	50.53 ± 9.01	48.68 ± 20.31	27.96 ± 8.71	38.22 ± 9.49	86.02 ± 14.50	12.93 ± 21.79	49.47 ± 8.14	54.62 ± 15.64	49.12 ± 12.43	51.87 ± 5.13
105	25	87.70 ± 8.09	18.49 ± 23.72	53.09 ± 10.34	47.76 ± 13.56	42.26 ± 14.95	45.01 ± 9.05	87.27 ± 7.53	14.07 ± 18.63	50.97 ± 9.68	45.38 ± 11.19	61.03 ± 15.14	53.21 ± 5.65
	50	87.00 ± 8.24	17.26 ± 19.61	52.13 ± 8.11	49.57 ± 19.65	36.05 ± 8.89	42.81 ± 9.19	85.92 ± 9.27	17.36 ± 19.37	51.64 ± 11.06	48.10 ± 7.16	58.45 ± 5.06	53.28 ± 3.60
	75	81.04 ± 10.83	11.97 ± 15.48	46.50 ± 5.81	48.39 ± 5.75	41.23 ± 19.12	44.81 ± 9.84	82.76 ± 12.37	18.81 ± 27.21	50.78 ± 13.79	55.93 ± 12.89	52.30 ± 9.71	54.12 ± 4.80

**Table 2:** Statistical difference of the percentage of muscle activation time in the cycling phases among workloads

Saddle height (%GTH)	Workload (%FTP)	Rectus femoris	Tibialis anterior	Biceps femoris	Medial gastrocnemius
95	25 vs. 50	NS	NS	NS	NS
	25 vs. 75	NS	NS	*	a
	50 vs. 75	NS	NS	NS	NS
97	25 vs. 50	NS	NS	NS	NS
	25 vs. 75	NS	NS	NS	NS
	50 vs. 75	NS	NS	NS	NS
100	25 vs. 50	NS	*	NS	NS
	25 vs. 75	b*	NS	NS	a b
	50 vs. 75	NS	NS	NS	NS
103	25 vs. 50	NS	NS	*	NS
	25 vs. 75	*	b*	NS	NS
	50 vs. 75	NS	NS	NS	b
105	25 vs. 50	NS	NS	NS	NS
	25 vs. 75	*	NS	NS	a
	50 vs. 75	*	NS	NS	a

Statistical difference when *p* < 0.05

*NS* non-significant

a indicates a statistical difference in the propulsive phases

b indicates a statistical difference in the recovery phase

*indicates a statistical difference in a complete crank cycle

## Discussion

The purpose of our study was to investigate the effects of saddle height and cycling workload on the muscle activation of the lower limb muscles (RF, TA, BF, and MG). The first primary hypothesis was that the EMG signals of these four muscles would be affected by saddle height, but the trend of change could not be consistent. This hypothesis was only partially supported, as only the EMG of MG demonstrated a statistically significant increase as saddle height increased. The second main hypothesis was that the EMG signals would increase as the cycling workload increased. Only the RF and BF were accommodated with this assumption, and EMG signal changes in the TA and MG were not statistically different. The pattern of muscle coordination may not change since there were no appreciable changes in the onset/offset timing and duration of muscle activation.

Cycling mainly relies on the lower limb muscles to propel the cyclist forward. The EMG signal provides information on the electrical activity of the muscle fibers during contraction. The amplitude of the EMG signal reflects the pattern of muscle activation, number of motor units recruited, and their firing rates, while the mean of EMG signal reflects the activation level during a crank cycle [17, 18].

### The impact of cycling workload

Cycling workload appears to have a greater effect on the activation state of lower limb muscles than saddle height. The results showed an increasing trend in the EMG signals of the RF and BF with increasing cycling workload, which is consistent with previous studies. Sarre et al. [10] found that regardless of the freely chosen cadence the means of normalized EMG of RF, VM, and VL were the highest at the maximum workload. The 60%, 80%, and 100% of maximal aerobic power as the workloads and a different normalizing approach used in their study might contribute to larger EMG than our results. The effect of workload on RF was consistent with our study. An outdoor cycling study found that workloads were associated with increased levels of muscle activity, among which RF and VL were the muscles most responsible for higher power outputs [6]. The validation in outdoor cycling experiments extends the universality of our findings, although the available data in the analysis were limited due to the adverse effects of the outdoor environment on data gathering. In an incremental cycling experiment on the effects of preferred legs, the activation of VL and BF in both the preferred and non-preferred legs increased considerably with increased exercise intensity expressed in watts [19]. However, the studies exploring the effect of workload on the activation of BF are fewer. Consequently, the results of BF collected in this study provide a crucial addition to reveal the activation of lower limb muscles.

Participants in this study were asked to keep the cadence between 85 and 95 rpm throughout the experiment. When the workload increased, more power was needed to overcome the resistance and maintain a constant pedaling speed. The total mechanical work demands for cycling increased, which modulated muscle activation and the number of motor units recruited, especially during the propulsive phase [20]. Approximately 39% of the total positive mechanical work was produced by the knee extensor muscle, accounting for the highest proportion [21]. The RF is one of the quadriceps muscles and plays an important role in extending the knee and flexing the thigh. Therefore, it is expected that the EMG signal of RF increased significantly with the increase of cycling workload. Hip extensors are the muscle group that produces the second most mechanical work after knee extensors, accounting for 27% of the total positive mechanical work [21]. The BF connects the femoral trochanter with the epicondyle of the tibia and the tip of the fibula, assisting in hip extension and knee flexion. Therefore, it is reasonable that BF had a positive response to the increase in workloads and improved the activation level. On the other hand, increasing cycling workload leads to changes in muscle fiber recruitment patterns [22]. During low-intensity cycling, slow-twitch muscle fibers were primarily recruited. As the workload increased, fast-twitch muscle fibers were also recruited to generate more force [23]. Fast-twitch muscle fibers have a higher firing rate, leading to an increase in the amplitude of the EMG signals. Moreover, there is a shift in muscle activation patterns from concentric to eccentric contractions. During the recovery phase, the RF undergoes an eccentric contraction, while the BF undergoes a concentric contraction. During the propulsive phase, the RF and BF undergo concentric contraction and eccentric contraction, respectively [24]. Eccentric contractions produce more force than concentric contractions, which enables the muscles to withstand a greater workload [25]. As a result, when the cycling workload increased, the eccentric contractions became more prominent and then led to an increase in the amplitude of the EMG signals.

In summary, an increased number of motor units recruited, and their firing rates are related to muscle activation and a shift in muscle fiber recruitment patterns, which could explain the increasing amplitude and mean values of the EMG signals of the RF and BF.

The vastus medialis (VM), RF, BF, MG, and soleus muscles in the previous study, with the exception of the TA, showed higher total muscle activation in response to an increase in workload [22]. This confirmed the results of RF, BF, and TA in our study, while the results of MG seemed to be opposite. It is noteworthy that they recruited competitive cyclists and applied more workload increases compared to our study. They also reported that the rise in MG activation was significantly less than that of BF. Additionally, the activation of MG can be inversely related to workload during prolonged exercise as an attempt to postpone fatigue [26]. Therefore, the almost unchanged activation of MG with increased workload is reasonable in a short time cycling. The difference in workload increment might be the main reason for the disparate outcomes. One study [27] on semi-reclined cycling raised the workload from 0 to 100 W, resulting in the almost unchanged activation of MG and increased activation levels of BF and RF, which were in agreement with our study. But the EMG peak magnitude of TA increased by about three times. The main distinctions from our study are their initial workload and the bicycle type. Although TA muscle is activated mainly during the recovery period [28], semi-reclined cycling modified its peak timing and duration of activation and improved its co-activation with RF. This might be the reason for the disagreement of TA results. In normal upright cycling, TA acting mainly in recovery period contracts isometrically to stabilize the ankle joint and maintain the foot position on the pedal [28]. The function of the ankle stabilizer and dorsiflexor might account for the absence of alterations in the EMG signal of the TA in our results [29]. The co-activation of the TA and MG was associated with knee flexion during the recovery phase and ankle stability during the propulsive phase, which suggested that the cause for the TA may also be the explanation for the unchanging EMG results of the MG [30]. The TA and MG are not the primary muscles responsible for generating power during cycling [31]. The quadriceps muscle group, including the RF, contributes to the highest percentage of total power, followed by hamstring muscles, including the BF [31, 32]. Therefore, the EMG activations of the TA and MG did not change significantly with increasing cycling workload. Additionally, the TA and MG are endurance muscles that are fatigue resistant and have a high oxidative capacity. This enables them to maintain a constant level over a long time, even during high-intensity cycling [33]. In addition, due to the smaller size of the TA and MG muscles compared with the quadriceps and hamstrings, they are limited in the number of recruitable motor units [34–36]. Therefore, the reason why the EMG signals of the TA and MG were almost unchanged might be explained by their duties in cycling, period of activation, and smaller muscle size.

### The impact of saddle height

The finding that the EMG signals of the MG were significantly affected by saddle height is consistent with previous research. The MG increased dramatically in integrated EMG value with higher saddle height (105% of preferred height) compared with the self-selected height and lower height (90% of preferred height) [37]. In contrast to self-selected height, referring to anthropometric parameters is more objective and unbiased. One study compared the EMG signals of the lower limb muscles at saddle heights of 90% GTH and 100% GTH [38]. The EMG integral and maximum in the low-saddle condition were 65% and 62% of those in the high-saddle condition, respectively. Our study supports this result, which can be explained by the kinematics of the ankle and knee joints. The changes in joint angles resulted in alterations in the stretch–shortening cycle and lengths of muscles [39]. As the saddle height increases, the flexion angle of the knee decreases and the extension angle increases [40]. The ankle exhibits the opposite, with an increase in dorsiflexion [41]. The increased demand for dorsiflexion torque required at higher saddle heights might lead to a larger maximum and mean EMG signal of the MG [42]. The lengthening velocity of the MG increased during the propulsive phase and decreased during the recovery phase as the saddle height increased [37]. The MG engaged in centrifugal contraction and centripetal contraction during the propulsive phase and recovery phase, respectively [43]. According to the force–velocity curve of muscle [44], the MG would generate more tension force throughout both phases.

In contrast, the lack of significant effects of saddle height on the EMG signals of the RF, BF, and TA in our study was different from previous studies [14, 45]. The duration of MG eccentric contraction decreased and that of BF increased with increasing saddle height in a previous study [14]. This is the same as the results in our study when the saddle height was increased from 95 to 100% GTH with a constant workload. However, the degree of change was minimal, and there was no statistically significant difference. This might be because the cycling workload in our study is much smaller than the 200 W used in their study [14]. However, our results are consistent with the findings of another study [45] that only the activation time of the VL muscle increased significantly with higher saddle height, and the EMG results of BF, RF, and gastrocnemius were not significantly different [45]. The main reason might be that the VL is a monoarticular and primary power producer [46]. Conversely, RF and BF are biarticular muscles, behaving differently and with greater variability in different cycling phases [8]. Therefore, saddle height might have an impact on muscle activation intensity, number of muscular units recruited, and muscle coordination pattern. This combined effect ultimately results in a minor change in the EMG signal. On the other hand, the findings of several statistically different studies lead to divergent conclusions. The level of muscle activity for RF and BF increased when the saddle was lowered to 95% of the optimal height [7]. However, others argued that the activities of the quadriceps (RF and VM) were not related to saddle height [21]. Contrary results have even been reported in which the activation of RF and BF muscles was reduced with lower saddle height [45]. These discrepancies could be easily explained by the variations in cycling workload applied and in the calculational strategies used for the saddle heights. The 106% of crotch height used as the neutral saddle height in a previous study [16] is close to the 100% GTH in our study [3]; hence, their results of the increased activation level of MG and unchanged EMG signals of TA with higher saddle height were consistent with ours.

An intriguing discovery is that, regardless of the cycling workload, the maximum and mean EMG values of RF at 100% GTH saddle height were lower than those in the other situations with higher or lower heights. A similar trend was observed for the EMG results of the BF when the workload was fixed at 25% FTP and 75% FTP. In addition, when the workload was 50% FTP, the EMG mean and maximum values of the 95% GTH condition were slightly smaller than those of the 100% GTH, but the latter was still smaller than the results of any other saddle heights. For the TA, the muscle activation level of 100% GTH was minimal for all heights only when the workload was 25% FTP. These results are sufficient to show that the saddle height of 100% GTH would have special significance. A more comfortable saddle height makes cycling easier, and less muscle activation is needed to cope with the same cycling workload [45]. Numerous studies on the ideal saddle height are based on anthropometric measurements. A range of 106–109% of the inseam length as the optimal saddle height during cycling was suggested [47]. This height range generally equates to 99–102% of the GTH [3]. Hamley and Thomas recommended use of 109% of inseam for optimal cycling performance [48]. Recent studies have shown that this height resulted in a large amount of variability in knee angle [49] and no advantage in cycling economy [50]. This may be because the lower limbs move periodically around the greater trochanter, and the inseam length is different from GTH. Therefore, the proposed GTH in this study may be more reliable. Although many studies begun to focus on the role of knee angle in bike fitting, joint angle seems to be more affected by riding techniques and other bike settings such as crank and frame [51]. And there is a 5° to 10° difference between static and dynamic joint angles [52, 53]. Therefore, bike fitting based on anthropometry is still the mainstream way and research focus. Some research proposed various formulas considering the linear and angular kinematics to predict the optimal saddle height [52, 54, 55], accounting for 54% of the relevant studies. Leg length is always a crucial variable in the formula, even though it has not been verified which formula is more reasonable. In contrast, only 17% of studies about saddle height have measured muscle activation [2].

Additionally, cycling economy was less affected when the saddle height varied between 96% and 100% GTH, even though muscle activation and technique were changed [37, 56]. Previous research has demonstrated that preferred saddle height leaded to the lowest peak power and muscle activation, with a 2.5% rise inducing a greater increase in muscle activation than a 2.5% decrease [45]. The distinction in the specific EMG values could potentially derive from individual variances and the fact that preference height is not equivalent to 100% GTH. Even so, both our results and theirs demonstrate that high saddle height is more likely to cause muscle fatigue. More direct validation came from self-reported results from 20 cycling club members who said the highest heights in the test were the most uncomfortable, with the highest levels of fatigue and pain in the thighs and knees [57]. It has been proposed that a 97% GTH minimized the average absolute hip and knee moments. However, the study only included three male trained cyclists. The cycling level of participants may have influenced the results. GTH was the reference used in two other studies. One study found that 96% to 100% GTH was the optimal height range for minimal VO_2_ [58]. Another study more specifically pointed out that 100% GTH minimized VO_2_ and adapted for knee and ankle joint kinematics [59]. However, the subjects of the studies were competitive cyclists and female cyclists, respectively, which differ from ours. The conclusions are still consistent with this study.

The experimental results showed that only the EMG signals of the MG muscle were significantly affected by saddle height, while the EMG signals of the other three muscles showed no significant differences at different saddle heights. The maximum and mean values of the EMG signals of the RF, BF, and TA were found to be smallest at the saddle height of 100% GTH with specific workloads. This might indicate that among the five tested saddle heights, 100% GTH is the most appropriate height.

### The onset/offset timing and duration of activation

The onset and offset timing of muscle activation refers to the time at which the muscle begins to contract and stops contracting during the pedal stroke, respectively [60]. In our study, there were few statistically significant differences in the changes in onset/offset timing and duration of muscle activation. However, Brian et al. indicated that RF and BF displayed an earlier burst onset as the workload increased [61]. Despite the lack of statistical difference, the similar changing trends of RF and BF were found in Fig. 5. Cadence was constant in our study, but it was their experimental variable, which has been proven to be a critical influential factor on the onset/offset timing and duration [62, 63]. The interaction between workload and cadence might amplify the changes in muscle activation patterns. On the other hand, a study involving both athlete and non-athlete cyclists supports our results [64]. There were no differences in the onset and offset timing between cycling tests for both athlete and non-athlete cyclists. They also controlled the cadence at 90 rpm. Neptune and Herzog [31] noticed negligible amounts of negative muscular crank torque created at 90 rpm, but greater cadence more than 105 rpm produced substantial negative effect related to the duration of muscle activation. Therefore, the steady activation timing and duration may be benefit by the small cadence. In an incremental cycling test [65], the onset timing of RF in the final stage was earlier than that in the initial stage, but there was no significant difference between the middle stage and the initial stage. Their unchanged results of BF activation timing were aligned with our study. The long-time cycling with increasing workload continuously might cause muscle fatigue. Two of their participants did abort the experiment because of fatigue. Therefore, the change in onset timing in the final stage was likely caused by fatigue. Our results were well supported by the unaltered activation timing of BF and RF in the medium stage, when the participants were not tired.

There is no conclusive evidence, despite a report suggesting that saddle height would modify muscle activation timing [47]. The offset timing of RF, BF, and TA was statistically different but not different in onset timing, when the saddle height relative to the usual height was raised to leg fully extended or dropped to increased knee flexion conversely [66]. However, the preferred saddle height and leg length of participants were different, resulting in differences in the initial usual height and the degree of height variation. A more clear and uniform method for defining saddle height would be helpful to improve the reliability of the results. Another study supports our findings, the onset timing and duration of BF, MG, and VL were not significantly affected when saddle height was raised from 96 to 100% of GTH [14]. But they indicated that a later eccentric contraction offset of BF occurred with increased saddle height. It might be that the nervous system made adaptive changes to the timing of BF eccentric contractions to maintain the cycling efficiency at a constant cadence. However, they did not distinguish between contractile element and series-elastic element lengthening. Additionally, it is crucial to emphasize that variations in the definition of muscle activation thresholds significantly reduce the comparability of the outcomes between the studies.

### Practical implications

The research results of saddle height and workload may offer some practical suggestions on bike fitting, injury protection, and performance improvement to benefit coaches and cyclists.

A saddle height of 100% GTH may be beneficial in terms of reducing muscle activation and improving cycling economy. Cyclists and coaches can consider using this reference height as a starting point when adjusting saddle height. However, it is important to note that individual variations in anatomy, riding style, and comfort should also be taken into account. The substantial changes in MG muscle activation with different saddle heights highlight the importance of addressing muscle imbalances in cyclists. A low saddle height restricts the ability of the MG muscle to assist the soleus muscle in counteracting excessive dorsiflexion [67]. The under-activated MG muscle can lead to inadequate force transmission, compromised joint stability, and increased stress on other muscles or joints. These imbalances may potentially contribute to overuse injuries or joint-related issues, such as knee pain or Achilles tendinopathy. Therefore, optimizing saddle height can reduce the risk of these overuse injuries.

Considering that the RF and BF exhibited increased activation with higher cycling workloads, cyclists and coaches can strategically adjust the workload intensity to target the power muscles. Incorporating interval training or hill climbs that challenge these muscles can help improve their strength and endurance, leading to enhanced cycling performance. Simultaneously, cyclists need to consider the muscle ability before increasing the cycling workload during training. The power muscles, such as RF and BF, might be the first to develop muscular strain during high-workload training.

The results of onset/offset timing and activation duration suggest that the coordination and sequencing of muscle activation during the pedaling cycle remain relatively consistent across different cycling conditions. This consistency enables that cyclists could transfer their learned muscle coordination patterns to different cycling scenarios with minimal adjustment, which can enhance performance and reduce the risk of injury.

### Limitations and future research

The current study still had some limitations which could be refined in future studies. (1) The identical bicycle configuration, such as the height and reaching distance of the handlebar, might not satisfy participants’ particular needs. Although only the muscles of lower limb were studies, research has shown that the posture of the upper limb affects the activation of the lower limb muscles [68], a more comprehensive bike fitting should be performed before the experiment in future studies; (2) Only young healthy males with recreational cycling experience were recruited in our study. Thus, the findings might not be generalizable to professional cyclists and cyclists in other age groups. The female cyclists might have different cycling patterns because of the physiological and anatomical differences [69, 70]. Recruiting subjects of different age, gender, and cycling experience to understand how factors such as hormonal variations, body composition, and muscle architecture affect muscle activation and coordination; (3) Even though we have strengthened the fixation of EMG sensors using skin membranes and tapes, the EMG sensor might still shift as a result of the constant cycling movement; (4) The error of characterizing muscle activation directly with EMG signals should be considered due to the filtering effect of adipose tissue and the interference of surrounding muscles. To reduce this inaccuracy, we employed MVC to normalize the EMG signal [71]; (5) Due to the limited number of EMG sensors, we only tested four muscles of the right leg. Analyzing EMG signals from more muscles of both legs would be important to thoroughly reveal the coordination patterns of lower limbs; and (6) During the experiment, we used a minor cycling workload intending to reduce the influence of muscular fatigue, which might lead to the results of onset/offset timing not being significantly different. The larger workload should be investigated in future studies to gain a more comprehensive understanding of how low-limb muscles respond to increased demands and potential fatigue during cycling.

## Conclusion

The current study investigated the effects of cycling workload and saddle height on muscle activation and coordination patterns of key lower limb muscles (RF, TA, BF, and MG). When the workload ranged from 25 to 75% FTP and the saddle height ranged from 95 to 105% GTH, there were no interaction effects between the two variables. The muscle activation of the RF and BF increased with increasing cycling workload. The muscle activation of the MG was strengthened with higher saddle height, while the EMG signals of RF, TA, and BF were barely altered. This might be because the RF and BF are the power muscles generating the required mechanical work for cycling, while the MG, as a force transmission between lower limb joints, is influenced by the joint angle rather than the cycling workload. Therefore, cycling training with a higher workload may only benefit some specific muscles, such as the RF and BF. On the other hand, an appropriate saddle height could make cycling easier and reduce muscle fatigue and the risk of joint injury. The noteworthy finding is that when the cycling workload was fixed, the muscle activation of RF at the condition of 100% GTH was minimal among all saddle height conditions. The BF and TA also showed the most cases of minimal muscle activation at 100% GTH. This saddle height is probably appropriate for most cyclists. This study explains the mechanism of how cycling workload and saddle height affect the activation state of lower limb muscles by using EMG analysis from the perspective of lower limb biomechanics, which might have theoretically significant implications for reducing cycling injuries and enhancing competitive performance.

## Methods

### Participants and ethics

This study recruited healthy male participants from the Hong Kong Polytechnic University. All volunteers who signed up for the cycling experiment were asked to fill out a questionnaire (see the Additional file 1) before being included in the experiment about their personal information, cycling experience, and medical history. The inclusion criteria were between 20 and 30 years old, having a BMI between 19 and 24 kg/m^2^, and a height between 165 and 180 cm. The exclusion criteria were no cycling experience, cycled frequently less than once per week, rode less than 10 min on average each time, had been diagnosed with any musculoskeletal disease in the past 6 months, and had any knee pain in the past 6 months. Finally, eighteen healthy male participants with recreational cycling experience participated in this study. They were all self-reported to have some cycling experience or to be amateur riders, and they were free of lower extremity injuries and pain from the previous 6 months. The detailed participant information can be found in Table 3.

**Table 3:** Subject characteristics

Parameters	Participants (n = 18, mean ± SD)
Gender	Male
Age (years)	24.39 ± 2.75
Height (cm)	175.92 ± 3.92
Weight (kg)	68.51 ± 6.51
BMI (kg/m^2^)	22.13 ± 1.92
Greater trochanter height (cm)	89.44 ± 3.32

In a power analysis for a repeated measures analysis of variance, a sample size of 16 was estimated in G*power 3.1.9.7 [72] with a small effect size of 0.25, a β level of 0.8, and a α level of 0.05. The participants were informed of the experimental protocols and precautions before the experiment. Then, they were required to read and sign an informed consent form approved by the University Human Subjects Ethics Sub-Committee (Number: HSEARS20220615001).

### Instruments

**Fig. 6:**
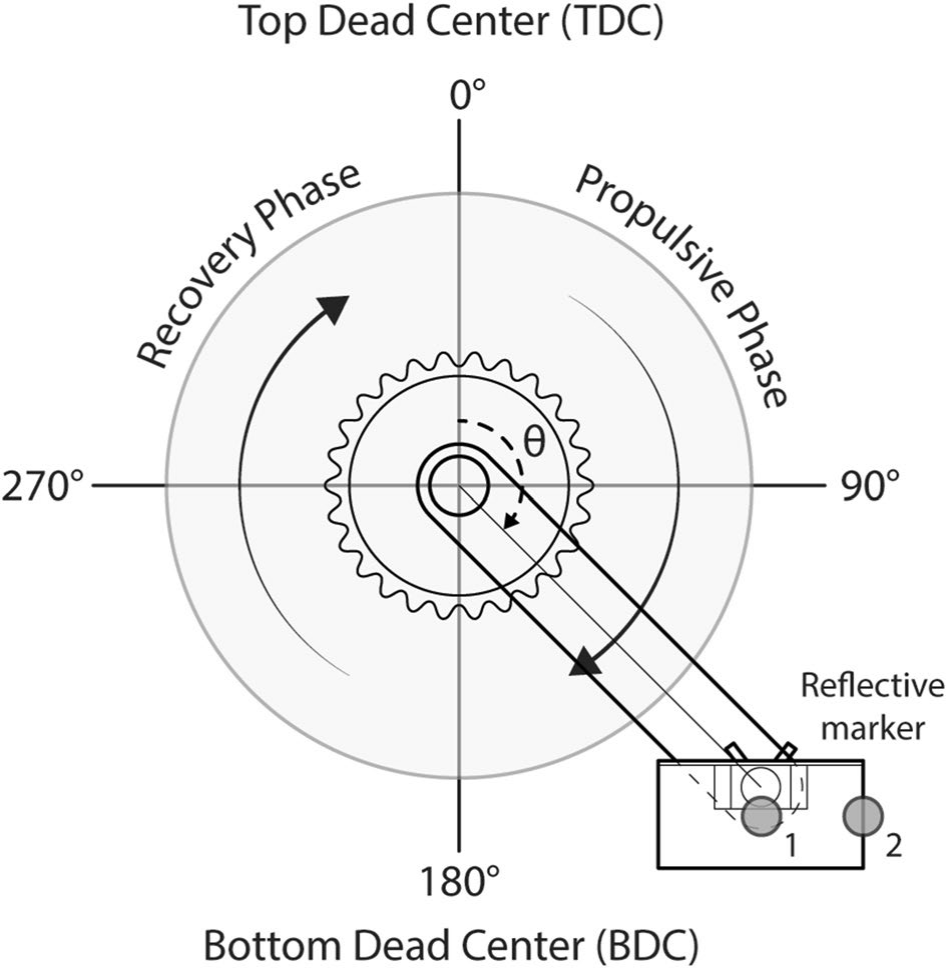
Schematic illustration of the right pedal. The propulsive phase is from 0° to 180°, and the recovery phase is from 180° to 360°. The gray spheres indicate the location of the reflective marker 1 and marker 2

An M-size mountain bike (Marlin 7 Gen 2, Trek, Intrepid Corporation, USA) and a smart cycling trainer platform (Tacx NEO 2T, Garmin, USA) were assembled for the experiment. The trainer platform allowed a precise workload setting. The handlebar position was uniform. The crank length was 170 mm. The saddle was adjusted (upwards/downwards and anteriorly/posteriorly) to fit each participant in the trial riding stage, while the saddle height, as an experimental variable, was set according to the height of the participant's greater trochanter to the ground (GTH) in the formal experiment. The gear ratio was fixed at 1.733 (26T for front chainring and 15 T for rear wheel) for all participants. To locate the pedal position and identify the cycling phase, one reflective marker (marker 1) was placed at the center of the outside edge of the pedal and another (marker 2) at the center of its front edge. Each pedal had two makers attached, as shown in Fig. 6. An eight-camera motion analysis system (Vicon Motion Analysis Inc., UK) was used to acquire trajectories of markers during the cycling tests at 250 Hz. Participants were required to wear uniform, tight-fitting tracksuits. The sole of the participant’s sneaker was required not to exceed 3.5 cm.

The surface EMG signals of the RF, TA, BF, and MG of individual right lower limbs were recorded using a wireless EMG system (Delsys, Boston, MA, USA) at a sample rate of 2000 Hz. After the skin on the surface of the muscle was shaved and cleaned, EMG sensors were attached to the belly of selected muscles according to the surface EMG for non-invasive assessment of muscles (SENIAM) guidelines [73].

### Experimental protocols

The height, weight, and GTH of the participants were measured, and body mass index (BMI) was calculated. The participants warmed up and stretched for 10–15 min after changing the uniform sports clothes. According to the instruction, they performed several stretches and hold each for 20–30 s and warm-up cycling at a slow pace. Then participants performed a 4-min functional threshold power (FTP) test to estimate their fitness. The definition of FTP is the highest power that a rider can maintain in a quasi-steady state without fatiguing for approximately one hour [74]. The conversion formula of the average maximum power output for 4 min and 60 min is [75] ${P}_{4 min}\times 0.75={P}_{60 min}$. Participants rested for at least 10 min after completing the FTP test.

Following the SENIAM clinical recommendations, EMG electrodes No. 1 to No. 4 were attached to the midpoint of the RF, TA, BF, and MG muscle belly of the right leg parallel to the muscle fiber in sequence. Each muscle underwent the maximum voluntary contraction (MVC) test employing the designated posture in SENIAM. The same experimenter applied resistance for the MVC test and required the participants to maintain each posture for at least 5 s.

The variables in the experiment were saddle height and cycling workload. Saddle heights were set to 95%, 97%, 100%, 103%, and 105% of the GTH. The participants rode in three workload conditions (25%, 50%, and 75% FTP) under each saddle height condition. In total, the participants pedaled in 15 cycling conditions (five saddle heights × three cycling workloads). Cycling was performed for 2 min under each condition within a required cadence range between 85 and 95 revolutions per minute (rpm). The order of the testing conditions was randomized. Between conditions, participants had a minimum of two minutes of rest. EMG signals of four muscles of the right leg were recorded throughout the experiment.

### Data processing

The final one minute of each two-minute cycling test was used to collect data, which included at least five consecutive and complete pedaling cycles. A complete pedaling cycle of the right limb was defined from the top dead center (TDC, 0°) to ending at TDC (360°), which contained the propulsive phase and recovery phase, as shown in Fig. 6. The raw EMG signals were filtered using a zero-lag 4th-order bandpass Butterworth filter with cut-off frequencies of 10–500 Hz to remove noise and artifacts. The filtered EMG data were full-wave rectified to obtain the absolute value of the signal, and then further processed using a moving root-mean-square (RMS) with a moving window of 70 ms to obtain an envelope. The same process was done for four EMG signals from the MVC test. The resulting envelope was averaged to get the MVC values for the four muscles. The processed EMG signals were normalized to the MVC values for the respective muscle. The 3D motion files of the experiment were opened with Nexus software. The selected time periods for EMG analysis were intercepted and kinematics of markers were exported in excel file. The Z coordinate of marker 1 was used to divide the riding cycles. TDC and bottom dead center (BDC) corresponded to the moments of the maximum and minimum of Z coordinate, respectively. Therefore, one cycle was the process of the coordinate value from the maximum to the minimum and then to the maximum. These time points were used to divide the normalized EMG signal into five segments, each of which contained a complete cycle. The final EMG envelope under each cycling condition for specific muscles was the mean of the five signals. The maximum and mean values of the EMG envelope were calculated for statistical analysis.

The threshold for muscle activation was defined as the mean EMG amplitude minus half of the standard deviation. The onset and offset timing of EMG for participants under different cycling conditions were the times when the processed EMG signal exceeded this threshold. The duration of activation is the time between the onset and offset timing.

### Statistical analysis

Two-way analysis of variance (ANOVA) was conducted to examine the effects of saddle height and cycling workload on the muscle activation level. Residual analysis was performed to test the homogeneity of variance and Shapiro‒Wilk's test was used to exam the normality for the assumptions of the two-way ANOVA [76]. None of the normalized EMG data showed any significant interactions of saddle height and workload (*p* > 0.05, ${\eta }^{2}$ = 0.010). Therefore, the separate one-way ANOVAs for each variable were conducted. The homogeneity of variance was assessed by Levene’s test, and the sphericity was checked by Mauchly's test [77]. Effect sizes are expressed as partial eta squared (*η*^2^), and they are classified as large (${\eta }^{2}$ ≥ 0.15), medium (0.06 ≤ ${\eta }^{2}$  < 0.15), and small (0.01  ≤ ${\eta }^{2}$  < 0.06) [78]. The analysis of the main effect for saddle height and workload was performed with reported 95% confidence intervals. Significance was set at $\alpha \le 0.05$ [79]. All statistics were conducted by SPSS (Version 26, SPSS Inc., Chicago, IL).

### Supplementary Information

**Additional file 1. **Cycling exercise questionnaire.

**Additional file 2: Table S1.** Mean values of electromyographic (EMG) activity of lower limb muscles.

**Additional file 3: Table S2.** Maximum values of electromyographic (EMG) activity of lower limb muscles.

## Data Availability

The datasets used in this study are available from the corresponding author on reasonable request.
