# Supplementary material for: Effects of workload and saddle height on muscle activation of the lower limb during cycling
Source: Biomed Eng Online. 2024 Jan 16;23:6. doi: 10.1186/s12938-024-01199-y (PMC10790431; doi:10.1186/s12938-024-01199-y)
Supplement: Supplementary file 1 — Additional file 1. Cycling exercise questionnaire. [file 12938_2024_1199_MOESM1_ESM.docx]

**Cycling Exercise Questionnaire**

The purpose of this questionnaire is to know your cycling experience and health status. All information is used for academic research only. Please answer according to the actual situation. Thank you for your kind assistance.

Part Ⅰ. Personal information

1. Name:
2. Gender: □ Male □ Female
3. Age:
4. Height (cm):
5. Weight (kg):

Part Ⅱ. Exercise regime

1. Cycling experience:

□ No experience □ Some experience □ Amateur cyclist □ Professional cyclist

1. Cycling frequency:

□ ≤ 1 time a week □ 2-3 times a week □ 4-5 times a week □ ≥ 6 times a week

1. Average time of every cycling:

□ ≤ 10 min □ 10-30 min □ 30-60 min □ 1-3 hr □ ≥ 3 hr

Part Ⅲ. Medical history

1. Have you ever been diagnosed with any musculoskeletal disease in the past 6 months? Yes / No

If yes, please briefly describe:

Diseases: _________________

Duration: _________________

1. Have you had any knee pain in the past 6 months? Yes / No

If yes, please briefly describe:

Degree of pain: ____________

Duration: _________________

Location: □ left knee □ right knee □ both knee

□ the front □ the back □ the left side □ the right side

Date:

**騎行運動調查問卷**

本問卷的目的是了解您的騎行經驗和健康狀況，所有資料僅供學術研究使用。請根據實際情況回答問題。感謝您的熱心協助。

1. 個人資料
2. 姓名：
3. 性別： □ 男 □ 女
4. 年齡：
5. 身高（釐米）：
6. 體重（千克）：
7. 運動習慣
8. 騎行經驗：

□ 無 □ 部分經驗 □ 業餘騎行愛好者 □ 專業騎手

1. 騎行頻率：

□ ≤ 1次每週 □ 2-3次每週 □ 4-5次每週 □ ≥ 6次每週

1. 平均每次騎行時長：

□ ≤ 10 分鐘 □ 10-30分鐘 □ 30-60分鐘 □ 1-3小時 □ ≥ 3小時

1. 患病歷史
2. 在過去的6個月中，你是否被確診患有任何肌肉骨骼疾病？ 有/沒有

如有，請簡述：

疾病名稱：______________

患病時長：______________

1. 在過去的6個月中，你是否感到過膝蓋疼痛？ 有/沒有

如有，請簡述：

疼痛程度：______________

疼痛時長：______________

疼痛部位： □ 左膝 □ 右膝

□ 前側 □ 後側 □ 左側 □ 右側

日期：
