# Supplementary material for: Effects of workload and saddle height on muscle activation of the lower limb during cycling
Source: Biomed Eng Online. 2024 Jan 16;23:6. doi: 10.1186/s12938-024-01199-y (PMC10790431; doi:10.1186/s12938-024-01199-y)
Supplement: Supplementary file 2 — Additional file 2: Table S1. Mean values of electromyographic (EMG) activity of lower limb muscles. [file 12938_2024_1199_MOESM2_ESM.docx]

**Table S1.** Mean values of electromyographic (EMG) activity of lower limb muscles.

| Saddle height (%GTH) | Workload (%FTP) | Rectus femoris (%MVC) | Tibialis anterior  (%MVC) | Biceps femoris  (%MVC) | Medial gastrocnemius (%MVC) |
| --- | --- | --- | --- | --- | --- |
| 95 | 25 | 9.19±7.60 | 6.96±4.01 | 10.73±7.58 | 10.58±5.76 |
|  | 50 | 10.12±6.70 | 6.93±4.26 | 11.96±7.86 | 9.23±6.54 |
|  | 75 | 13.38±9.66 | 8.14±5.74 | 14.64±9.15 | 10.50±7.33 |
| 97 | 25 | 8.83±7.17 | 7.83±5.62 | 11.60±9.94 | 12.54±8.60 |
|  | 50 | 10.48±7.93 | 6.62±4.17 | 12.93±8.69 | 10.87±7.43 |
|  | 75 | 12.40±11.51 | 7.65±5.50 | 14.60±10.03 | 10.95±8.27 |
| 100 | 25 | 7.13±5.52 | 6.79±5.03 | 10.29±8.81 | 11.00±6.87 |
|  | 50 | 9.32±6.97 | 7.04±4.68 | 12.58±9.68 | 12.48±7.36 |
|  | 75 | 11.66±6.86 | 6.79±4.35 | 14.59±10.90 | 10.73±6.86 |
| 103 | 25 | 9.72±9.75 | 7.22±4.45 | 14.16±12.37 | 12.75±6.33 |
|  | 50 | 10.44±8.23 | 6.22±4.34 | 14.54±2.98 | 13.44±7.79 |
|  | 75 | 11.74±8.11 | 6.73±5.34 | 16.41±12.20 | 15.00±10.07 |
| 105 | 25 | 10.15±9.91 | 7.31±4.15 | 14.77±13.03 | 16.53±7.46 |
|  | 50 | 10.30±7.35 | 6.82±4.56 | 15.00±11.99 | 18.71±9.56 |
|  | 75 | 12.67±10.55 | 7.21±4.38 | 17.04±12.27 | 16.15±7.51 |

Values are presented as Mean ± SD. Abbreviations: *GTH*, greater trochanter height; *FTP*, functional threshold power; *MVC*, maximum voluntary contraction.
