# Supplementary material for: Effects of workload and saddle height on muscle activation of the lower limb during cycling
Source: Biomed Eng Online. 2024 Jan 16;23:6. doi: 10.1186/s12938-024-01199-y (PMC10790431; doi:10.1186/s12938-024-01199-y)
Supplement: Supplementary file 3 — Additional file 3: Table S2. Maximum values of electromyographic (EMG) activity of lower limb muscles. [file 12938_2024_1199_MOESM3_ESM.docx]

**Table S2.** Maximum values of electromyographic (EMG) activity of lower limb muscles.

| Saddle height (%GTH) | Workload (%FTP) | Rectus femoris (%MVC) | Tibialis anterior  (%MVC) | Biceps femoris  (%MVC) | Medial gastrocnemius (%MVC) |
| --- | --- | --- | --- | --- | --- |
| 95 | 25 | 21.71±15.90 | 22.11±15.35 | 26.42±19.00 | 29.02±14.72 |
|  | 50 | 24.50±12.18 | 21.96±15.85 | 31.25±20.69 | 23.93±16.06 |
|  | 75 | 34.19±21.37 | 24.91±20.07 | 39.87±26.50 | 28.41±20.21 |
| 97 | 25 | 20.53±12.54 | 23.97±17.33 | 27.73±19.45 | 32.34±18.42 |
|  | 50 | 27.24±18.82 | 21.78±14.77 | 35.53±24.12 | 29.40±19.01 |
|  | 75 | 33.00±29.15 | 22.48±18.39 | 39.73±27.86 | 29.11±21.37 |
| 100 | 25 | 18.60±14.30 | 20.54±16.31 | 25.25±24.05 | 31.68±18.58 |
|  | 50 | 22.79±15.86 | 22.04±16.48 | 32.69±25.06 | 33.64±17.83 |
|  | 75 | 31.11±16.73 | 20.33±14.05 | 38.22±28.38 | 30.73±23.34 |
| 103 | 25 | 22.63±20.50 | 20.94±14.17 | 33.58±28.63 | 39.45±19.50 |
|  | 50 | 27.52±19.43 | 17.01±13.64 | 39.21±30.17 | 40.95±21.00 |
|  | 75 | 31.43±19.63 | 19.92±16.15 | 41.12±30.60 | 43.43±26.16 |
| 105 | 25 | 22.80±18.46 | 20.74±13.42 | 34.43±29.63 | 45.63±17.20 |
|  | 50 | 29.62±19.48 | 19.21±13.52 | 37.49±28.41 | 54.31±26.25 |
|  | 75 | 34.23±27.41 | 21.75±15.94 | 43.82±28.41 | 52.35±21.56 |

Values are presented as Mean ± SD. Abbreviations: *GTH*, greater trochanter height; *FTP*, functional threshold power; *MVC*, maximum voluntary contraction.
